# Spatial versus Day-To-Day Within-Lake Variability in Tropical Floodplain Lake CH_4_ Emissions – Developing Optimized Approaches to Representative Flux Measurements

**DOI:** 10.1371/journal.pone.0123319

**Published:** 2015-04-10

**Authors:** Roberta B. Peixoto, Fausto Machado-Silva, Humberto Marotta, Alex Enrich-Prast, David Bastviken

**Affiliations:** 1 Biogeochemistry laboratory, Department of Ecology, Universidade Federal do Rio de Janeiro, 68020, Rio de Janeiro, Brazil; 2 Sedimentary and Environmental Processes Laboratory (LAPSA/UFF), Department of Geography, Institute of Geosciences, Universidade Federal Fluminense, Niteroi, Brazil; 3 Department of Thematic Studies—Environmental Change, Linköping University, 58183, Linköping, Sweden; University of Wisconsin Milwaukee, UNITED STATES

## Abstract

Inland waters (lakes, rivers and reservoirs) are now understood to contribute large amounts of methane (CH_4_) to the atmosphere. However, fluxes are poorly constrained and there is a need for improved knowledge on spatiotemporal variability and on ways of optimizing sampling efforts to yield representative emission estimates for different types of aquatic ecosystems. Low-latitude floodplain lakes and wetlands are among the most high-emitting environments, and here we provide a detailed investigation of spatial and day-to-day variability in a shallow floodplain lake in the Pantanal in Brazil over a five-day period. CH_4_ flux was dominated by frequent and ubiquitous ebullition. A strong but predictable spatial variability (decreasing flux with increasing distance to the shore or to littoral vegetation) was found, and this pattern can be addressed by sampling along transects from the shore to the center. Although no distinct day-to-day variability were found, a significant increase in flux was identified from measurement day 1 to measurement day 5, which was likely attributable to a simultaneous increase in temperature. Our study demonstrates that representative emission assessments requires consideration of spatial variability, but also that spatial variability patterns are predictable for lakes of this type and may therefore be addressed through limited sampling efforts if designed properly (e.g., fewer chambers may be used if organized along transects). Such optimized assessments of spatial variability are beneficial by allowing more of the available sampling resources to focus on assessing temporal variability, thereby improving overall flux assessments.

## Introduction

Methane is an important greenhouse gas (GHG) that accounts for approximately 20% of the radiative forcing, according to a 100-year time period, and the global warming potential of CH_4_ is approximately 25 times greater by weight in comparison with carbon dioxide (CO_2_) [1]. Methane is primarily formed by a terminal degradation step in anaerobic degradation of organic matter by methanogenic archea [2–4]. Acetate or molecular hydrogen (H_2_) and CO_2_, being formed in earlier fermentative degradation steps are considered to be the most important substrates for methanogenesis, although the use of a handful of other small organic molecules have been demonstrated as well [2]. When reaching aerobic environments, CH_4_ can be oxidized to CO_2_ and water by methane oxidizing bacteria. Methane can also be oxidized under anaerobic conditions by syntrophic consortia using e.g. sulfate as electron acceptor [5]. As a consequence of these production and consumption pathways, high natural methane emissions can be expected from environments in which gases produced in anoxic zones can be transported to the atmosphere rapidly enough to escape complete oxidation before reaching the atmosphere.

CH_4_ emissions from lakes contribute significantly to the natural CH_4_ flux into the atmosphere but have until recently rarely been recognized [6]. Hence, lake emissions may offset current continental GHG budgets [7]. Methane can be emitted by multiple pathways from lakes, including evasion of CH_4_ dissolved in surface waters by diffusive flux, bubbling of CH_4_ from sediments (ebullition), and transport from sediments via rooted plants [8]. In many of the studied systems ebullition has dominated the open water fluxes, while plant mediated fluxes are large in systems where emergent rooted aquatic macrophytes are abundant [2].

Lake CH_4_ fluxes can be assessed in several ways but in general the monitoring gas accumulation in flux chambers has dominated so far. This methodology is conceptually simple and does not require expensive field equipment but is laborious by so far primarily relying on manual sampling. Given limited prior interest in freshwater CH_4_ emissions and the labor demanding measurements, the data collection approaches have not been yet systematically addressed spatiotemporal variability. Instead, CH_4_ flux data for lakes have often been characterized by non-repeated, short-term measurements, and a number of floating chambers are typically deployed for 30 minutes to 24 hours only. It is thus necessary to determine the representativeness of such “snapshot” measurements in addition to developing improved and optimized approaches to flux study design.

Because tropical, aquatic environments produce the highest volumes of CH_4_ emissions per area unit (e.g., fluxes m^-2^) [9] and cover large areas over the earth’s surface, the extrapolation of tropical freshwater flux values is of importance to the global CH_4_ budget [7]. In general, tropical lakes have high metabolic rates due to the high temperatures [10,11]. Many tropical lakes are situated in the floodplains surrounding the large tropical rivers. These tropical floodplain lakes have a varying depth depending on the hydrological season in the area and receive large amounts of organic material from the surroundings during periods of rising water [12]. Due to the large differences between high and low water levels in many tropical floodplains the aquatic macrophytes in floodplain lakes have adaptions to survive flooding. Many common macrophyte species are floating instead of being rooted and form macrophyte covered areas in the lakes. The macrophytes also contribute carbon to the lakes. Thus, tropical floodplain lakes receive large amounts of organic matter, which are rapidly converted to greenhouse gases including CH_4_ in the sediments [4]. Due to the relatively small change in temperature in tropical areas, the temperature stratification is not very strong and the tropical floodplain lakes mix frequently. Lakes of this type are also typically shallow (during large parts of the year, e.g. the low water periods); both these characteristics facilitating transport of GHGs from sediments and bottom waters to the atmosphere.

Given the high fluxes from tropical floodplain lakes, validations of the representativeness of previous measurements on such lakes are needed. As a first step towards addressing the representativeness of measurements of open water fluxes (i.e. diffusive flux and ebullition), we here address fundamental flux study design questions such as the number of chambers used, the spatial distribution of chambers, and day-to-day variability in flux in a tropical flood plain lake in the Pantanal of Brazil. The lake was studied intensively over five consecutive days, during which 29 flux chambers in different lake habitats were monitored repeatedly.

## Materials and Methods

### Study Area

The Pantanal is the largest floodplain in South America [13,14]. It is situated along the Paraguay River and the inundated area varies between 10,000 and 110,000 km^2^ during low- and high-water periods, respectively (data from 1979–1987) [14]. The floodplain is characterized by high summer season precipitation [14]. The annual inundation pulse distributes significant terrestrial inputs of organic and inorganic matter into warm, shallow waters [14]. These conditions favor oxygen depletion and extensive CH_4_ production and emission [4,15,16]. Previous studies have confirmed a high CH_4_ flux from the Pantanal, but have been limited through the use of short measurement periods and a limited number of chambers [9,17].

**Fig 1:**
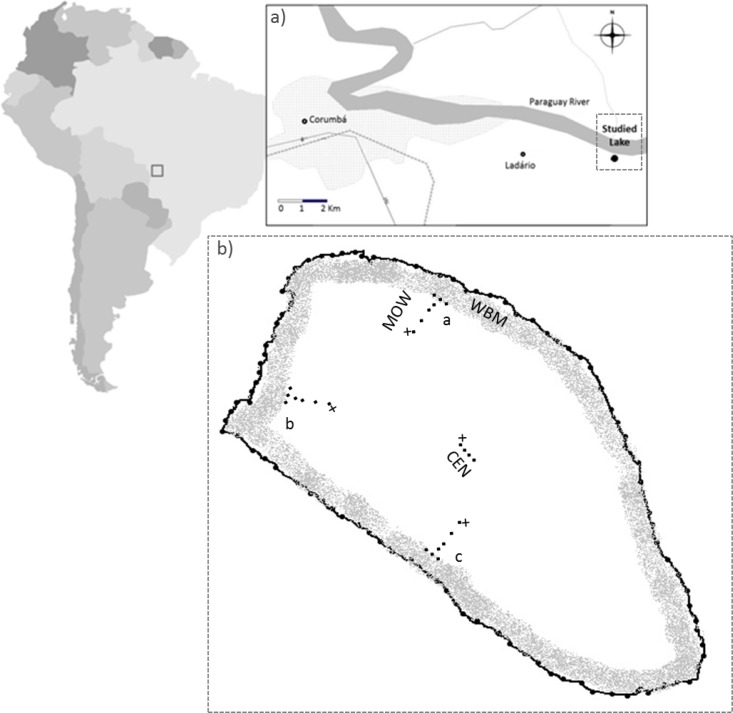
Geographic location of the studied lake (a). Chamber distribution in the studied lake (b). WBM denotes chambers in margin waters between emergent aquatic macrophytes (n = 3 chambers per transect a, b and c). MOW includes chambers in the open water area (positioned approximately 5, 10, 20 and 45 m from the macrophyte edge (n = 4 chambers per transect a, b and c). CEN represents chambers at the center of the lake (n = 4 chambers). Diffusive chambers that were equipped with a submerged bubble shield to prevent bubbles from reaching the chamber are denoted by crosses.

We studied a small shallow floodplain lake (depths varied spatially between 0.6 to 2.4 m at the time of the present study) that is situated close to the Ladário municipality (Province of Mato Grosso do Sul, Brazil, Fig 1A), where no specific permissions were required for the performance of research activities. The sampling work was developed by Brazilian researches in a public area in Brazil. We studied the spatial and temporal variability of air-water methane fluxes, and this study did not involve any endangered or protected species. The lake examined receives most of the water inputs from the Paraguay River and its tributaries during high-water periods (June and July). This lake is classified as eutrophic according to Salas and Martino [18].

### Study Design and Analytical Methods

Total CH_4_ fluxes from the lake to the atmosphere were measured using 8 L polyethylene floating chambers over 24-hour cycles for a period of five consecutive days during the low-water period of the dry season (Noon September 13 to noon September 18, 2008). The used chamber type do not bias diffusive flux [19,20]. Gas samples were collected through a syringe both immediately after placing the chambers into the water and once again after a period of 24 h to avoid bias from diel variability [9]. For ease of sampling, a 30 cm PVC tube (5 mm OD and 3 mm ID) was installed with one end inserted through a butyl rubber stopper at the top of each chamber and with the other end connected to a 3-way luer-lock syringe valve. The samples were transferred into 60 mL glass bottles that were completely filled with saturated salt-water solution and capped with gas-tight, 10-mm thick butyl rubber septa (Apodan, Denmark) prior to sampling. Samples were collected from the chambers through a 60 mL syringe and then injected into bottles positioned upside-down while allowing the brine to escape through a venting needle while the gas sample was injected. After this procedure, the gas sample constituted the headspace in the vials. Methane concentrations in the headspace were measured via gas chromatography using a flame ionization detector (GC-FID; Shimadzu GC-8, Poropack N column) in a laboratory. Detailed sampling procedures and calculation of the flux across the water surface and into the floating chamber, based on the net increase of CH_4_ over time, were described in Bastviken et al. [9].

The chambers were distributed across three lake zones: 1) margin water in the macrophyte belt—chambers were placed between emergent aquatic macrophytes (dominated by *Eichornia* sp.) close to the shoreline (WBM = Water Between Macrophytes; n = 9 chambers; water depth 0.6–1.2 m), 2) lake margin open waters, i.e., outside of but close to the macrophyte belt (MOW = Margin Open Water; n = 12 chambers; water depth 1.2–2 m), and 3) at the center of the lake (CEN = Centre; n = 4 chambers; water depth 2.4 m). The WBM and MOW zones were sampled along three transects (a, b and c; Fig 1B). The distance from the shore and the outer edge of the Eichornia belt varied between 5 and approximately 50 m depending on the location on the lake. The WBM flux chambers in the vegetation belt were located approximately 2 m inside the vegetation from the lake side, which correspond to different distances from the shore in the different transects; approximately 3, 30 and 10 m from the shore in transects a, b and c, respectively. MOW chambers were placed at approximately 5, 10, 20 and 45 m from the edge of the macrophyte belt (Fig 1B). To assess diffusive flux only, one additional chamber per transect equipped with a bubble shield placed 50–70 cm below the water surface to prevent bubbles from reaching the chamber was deployed along each MOW and CEN transect (n = 4 chambers; this technique is described at length in Bastviken et al. [9]; Fig 1B). For practical reasons, diffusive flux chambers (i.e. the chambers coupled with bubble shields) could not be placed in the WBM zone (either this zone was too shallow or the vegetation were too dense and often connected with submerged plant parts potentially disturbing any underwater bubble shields). To assess total flux, a greater number of the normal floating chambers without bubble shields were used in the WBM and MOW zones compared to in the center (CEN) due to previous indications of much lower variability levels in central zones [9].

### Statistical Analyses

Log-transformed data on CH_4_ flux showed significant Gaussian distributions (Kolmogorov-Smirnov, p > 0.05). We employed a one-way ANOVA to test differences between CEN, MOW and WBM CH_4_ emissions. In addition, because zones WBM and MOW possessed comparable transects, we used a two-way ANOVA to investigate whether differences between days, transects (a, b and c) and zones exist. Additionally, we employed a t-test to compare diffusive emission levels in margin and center zones. All tests applied a significance level of p < 0.05 using the software GraphPad Prism 4.0 and Systat 13.

## Results

No rainfall occurred over the five consecutive sampling days, though water and air temperatures increased from 24.1 to 27.0°C and 20.0 to 25.4°C, respectively. In the following discussion we focus on the air temperatures which represent daily averages, while the water temperatures were only measured once daily at noon and therefore are not representative for the whole day. During the study period the wind speed and barometric pressure was 1–4 m s^-1^ and 1000–1004 hPa, respectively (Table 1). The wind speed variability was irregular having no consistent pattern. The lowest pressures were observed in the ending of the study period but overall the pressure changes were small.

**Table 1:** Physic parameters for each sampling day.

Date	Wind (m/s)	Pressure (hPa)	Air temperature (°C)
09/14/2008	3	1004	20.0
09/15/2008	2	1003	22.9
09/16/2008	4	1004	25.5
09/17/2008	3	1004	23.9
09/18/2008	1	1000	25.4

Note that average values for each chamber deployment periods are given. The deployments periods started at noon the day before the sampling data and ended at noon during the sampling date. Hourly data obtained from a nearby weather station (Corumbá A724; www.inmet.gov.br).

**Fig 2:**
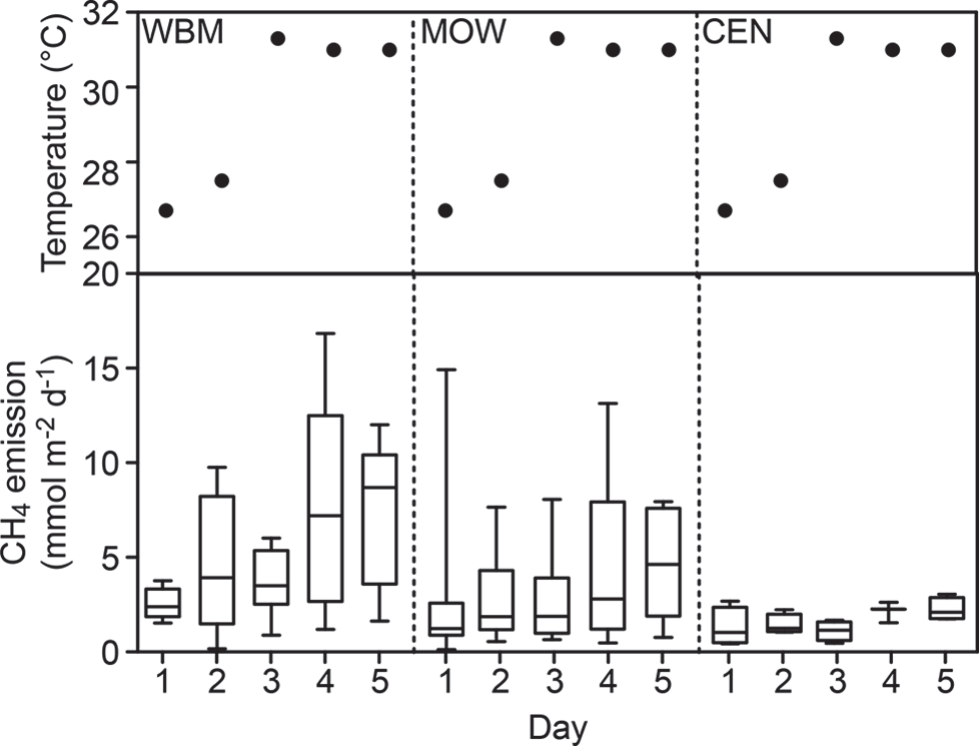
CH_4_ emissions per days. The upper panels show air temperatures over the course of the sampling period. The lower panels show lake CH_4_ emissions (mmol m^-2^ d^-1^) over five consecutive days in September of 2008 in the area between emergent macrophytes (WBM), the open water area (MOW) and the central (CEN) zone. Each bar represents a daily summary of all chamber measurements for each zone (see text for details). Bars show median values and 5–95% percentiles.

**Fig 3:**
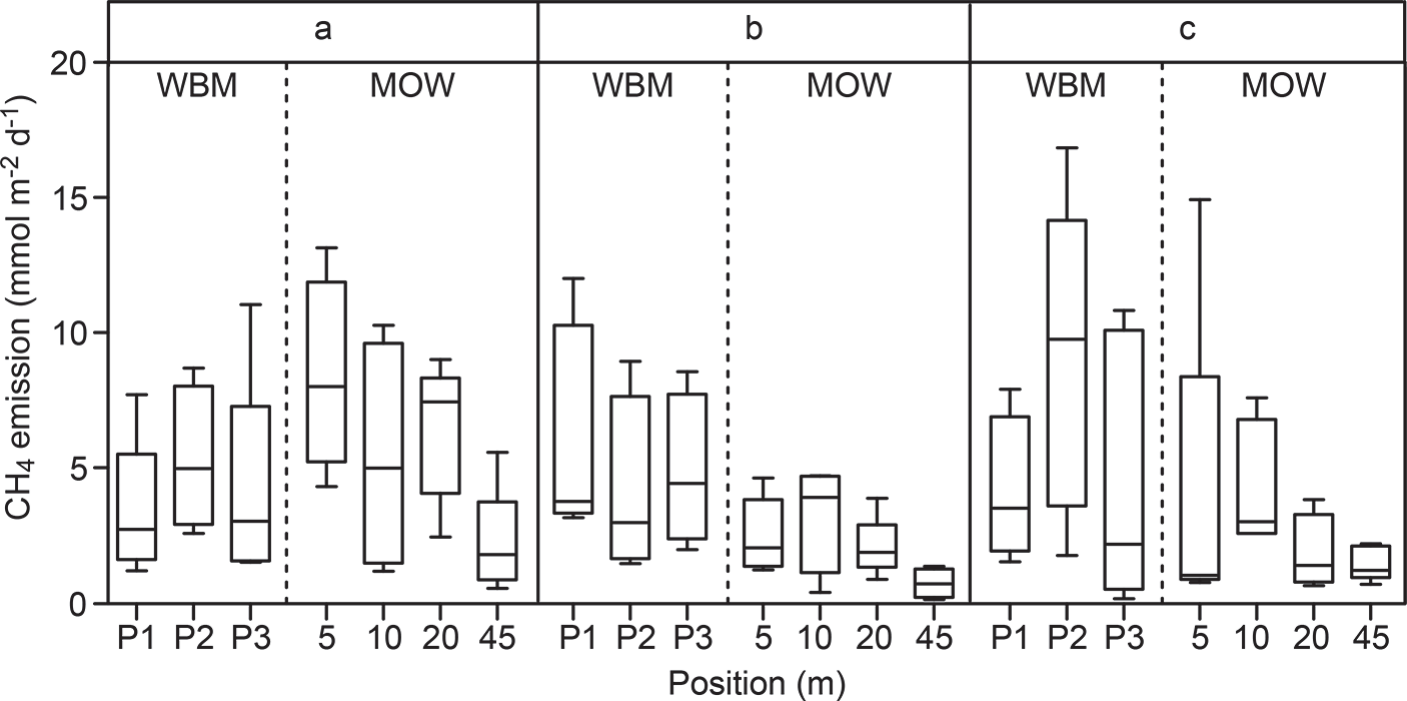
Comparison of CH_4_ emissions at each chamber position over all five days (mmol m^-2^ d^-1^) in WBM and MOW zones (see text). P1, P2 and P3 represent different chambers in the WBM zone and 5, 10, 20 and 45 m denote the distance from the macrophyte edge for MOW; both are plotted by transect (a, b or c). See Fig 1 for an illustration of chamber placement. Bars show median values and 5–95% percentiles.

Methane emission levels changed considerably within the lake with respect to both space and time (Figs 2 and 3 show collected data and Table 2 shows statistical test results). Average and median methane emissions approximately doubled between the first and fifth day (from 2.5 and 2.2 mmol m^-2^ d^-1^ on day one to 5.2 and 4.3 mmol m^-2^ d^-1^ on day five; Fig 2 and Table 2). For each zone separately, the air temperatures increase by 5.4°C between the first and fifth day, corresponded to a 198%, 48% and 74% increases in average WBM, MOW and CEN CH_4_ emissions, respectively. These flux changes were statistically significant for WBM and MOW, but not for CEN (2-way ANOVA; p = 0.02, 0.04, and 0.12, respectively). However, no significant differences were found when comparing day-to-day variability between consecutive days.

**Table 2:** ANOVA analysis results for CH4 emissions from the studied lake.

Questions	Answer	p<0.05
Differences between days?	Day 1 < Day 5. Otherwise no difference.	Yes
Differences between WBM, MOW and CEN zones?	WBM = MOW; WBM>CENT; MOW = CEN	Yes
Differences between transects within zones?	No	No

WBM, MOW, and CEN denote three lake zones, which representing water between plants in the hear shore floating macrophyte belt, the marginal open water area near the vegetation, and the center, respectively (see Fig 1). The MOW chambers were distributed along transects and showed decreasing flux levels from the edge of the macrophyte belt to the center, thereby including substantial spatial variability. Thus, the MOW flux was statistically similar to both WBM and CEN flux. See text for details.

**Fig 4:**
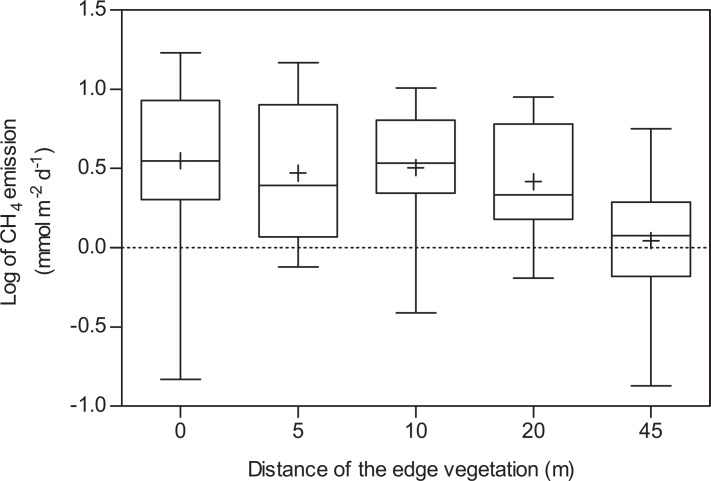
The effect of distance to the vegetation edge on CH_4_ emissions for WBM (plotted as 0 m) and MOW chambers (5, 10, 20 and 45 m). Bars show median values and 5–95% percentiles while cruses are mean values. Note the logarithmic y-axis scale.

Regarding spatial variations, no differences in CH_4_ emissions were found between transects (ANOVA, p>0.05; Fig 3 and Table 2). MOW emissions were analyzed from the edge of the macrophyte zone towards the center. Within the MOW zone, methane emissions were high in the range of 0–20 m from the macrophytes and then decreased. The emissions 45 m from the macrophyte zone edge resembled emissions in the CEN transect (Fig 4). Therefore, though MOW and CEN emissions were not statistically different, flux levels in the WBM zone were significantly higher than those recorded in the CEN zone. Higher degrees of variability were found for WBM (range 0.14–16.84 mmol m^-2^ d^-1^) and MOW (0.13–14.93 mmol m^-2^ d^-1^) emissions than for CEN emissions (0.43–3.03 mmol m^-2^ d^-1^). The median and average WBM CH_4_ emissions were 2.3 and 3.2 times higher than those generated in the CEN zone. In the same way MOW CH_4_ emissions were 1.3 and 2.2 times higher than those generated in the CEN zone, respectively (Fig 2; median and average values were 3.56 and 5.02 mmol m^-2^ d^-1^ in WBM, 2.04 and 3.48 mmol m^-2^ d^-1^ in MOW and 1.54 and 1.61 mmol m^-2^ d^-1^ in CEN, respectively). Diffusive flux accounted for an average of 12% of the total flux and was largely consistent across the margin and center (t test, p>0.05). Hence primarily ebullition, accounting for 88% of the total flux, was responsible for the spatial variability.

## Discussion

The diffusive flux represented a minor contribution to the total flux, especially in the WBM and MOW zones. High contributions of ebullition are consistent with previous results for small, shallow lakes [8,9,21]. It is important to note that measurements in the central lake parts only would have resulted in a severely biased emission estimate neglecting the large ebullition in the WBM and MOW zones. In the lake examined, CH_4_ emissions were on average 2.5 times lower than levels reported for the Pantanal [9,17] and Amazon floodplain lakes in previous studies [21,22]. This could be due to relatively low average temperatures during the sampling period (Table 1) compared to average Pantanal conditions. Overall, studies on CH_4_ emissions from *Eichornia* sp. areas report values between 0.36 mmol CH_4_ m^-2^ d^-1^ [23] and 20.9 mmol m^-2^ d^-1^ [9], highlighting the fact that variability over space and time is poorly constrained and that more studies are needed to fully understand CH_4_ production and emission dynamics in aquatic ecosystems.

Our study demonstrates that regardless of absolute flux values, greater and more variable CH_4_ emissions occur in marginal areas than in central areas (Fig 2 and Table 2). This pattern is further confirmed by the discovery of decreasing CH_4_ emissions between 20 and 45 m away from the vegetation in the MOW transects (Fig 4). Previous studies have identified strong relationships between water depth and emission levels, thus explaining variations in emission levels at different distances to the shore (e.g., distance to the sediment layer or variations in hydrostatic pressure influencing the release of ebullition) [8,17,24,25]. However, our results were not primarily correlated with water depth as the lake was shallow and the depth did not differ much between the edge of the macrophytes and the lake center. Other factors than depth may explain the relationship between CH_4_ emissions and distances to the shore in shallow lakes. In such lakes, this pattern is most likely attributable to a gradient in the organic substrate that may be used for methanogenesis in sediments depending on the distance from the macrophyte belt where most organic matter is produced by primary production. This observed spatial pattern may reflect the importance of organic substrates from littoral vegetation in the decomposition of organic matter [26–30], including methanogenesis in lake sediments [4,31].

The distinct pattern of decreasing flux with increasing distance from the shore demonstrates the importance of measuring emissions along transects from the shore for yielding representative emission estimates. However, comparing the different transects, no differences between them were found. Thus, although multiple transects are initially recommended for investigating spatial variability in each system, the results indicate that sampling efforts may subsequently be reduced to a small number of transects or even one single transect in shallow lakes with frequent and widespread ebullition.

While no significant differences were found with respect to consecutive day-to-day (24h) flux, changes in CH_4_ emissions between the first and fifth sampling day were found to coincide with a notable temperature change. Water levels and wind speeds did not change considerably over the 5-day period or did not vary in correspondence to the flux change. There was a small drop in pressure the last day (Table 1), that could have contributed to higher ebullition in the final day, but as the flux increase over time seemed to be gradual and follow the temperature (Fig 2). Changes in temperature were likely to contribute to this flux change. Methanogenesis is known to be highly sensitive to temperature [16,32]. Given exponential increases in CH_4_ formation rates in tropical lake sediments under a temperature increase from 20 to 30°C found by Marotta et al. [33], and given that most emissions generated from the studied lake were driven by ebullition, which may be directly related to CH_4_ production in the sediment, a flux increase is logical. The results may therefore illustrate that *in situ* CH_4_ flux (and not only CH_4_ formation rates observed in laboratory incubation settings) can be sensitive to temperature, as exhibited in other systems [34,35]. However, the observed increase of 48–198% among zones at a temperature change of 5.4°C is larger than expected and we cannot exclude that other the small pressure drop or other factors than those measured could have contributed.

This study provides evidence of high intra-ecosystem variability in CH_4_ emissions from a small low-latitude floodplain lake. The study also indicates that temporal variability may not only be driven by hydrology but also by weather-related variables. Thus, consideration of both spatial and temporal variability is critical. However, measurement efforts are resource demanding and it is important to balance efforts to the needs of covering both spatial and temporal variability. So far, spatial variability ha**s** often been prioritized compared to temporal variability (e.g. several chambers for spatial variability but measurements once or a few times only). Our results suggest spatial variability of the type of floodplain lakes studied here could be assessed through stratified sampling of key lake habitats and through the use of transects from the shore to central lake areas. Furthermore, although 24-h measurement campaigns may be representative of patterns that occur over time periods close to the sampling event, it is important to perform measurements under a range of weather and hydrological regimes to obtain representative flux estimates. We hypothesize that similar ways of organizing sampling efforts along transects based on distance to shore or depth could be used to cover spatial variability also in other types of systems as ebullition has been shown to vary with depth in deeper systems [8], although this remains to be tested. It should also be noted that other types of predictable spatial variability exist. For example it has been shown for water bodies having well defined inlet streams/rivers that CH_4_ emissions are often greater near the stream entrance, presumably due to a high sedimentation of particles carried by the stream resulting in intensive CH_4_ formation in the sediments locally [36].

Given the opportunity to address spatial variability with less sampling efforts once it is known or predictable, we suggest that, resources should be allocated to make the study of temporal variability should more extensive than those typically found in the literature thus far. This would contribute to more representative flux estimates in time, and importantly to improved possibilities to link fluxes to changes in environmental variables, in turn facilitating flux modelling and predictions. Our results thus provide guidance on ways to optimize available sampling efforts for examining spatiotemporal variability, which will be critical to the determination of representative flux estimates and to the future improvement of global greenhouse gas budgets.

## Supporting Information

S1 Table Data of methane emission from water to atmosphere. S1 Table describe the CH_4_ emission (mmol m^-2^ d^-1^) with respectively day, position and transect within lake. See Fig 1 in methods section for an illustration of chambers placements.
(PDF)
