# Supplementary material for: Spatial versus Day-To-Day Within-Lake Variability in Tropical Floodplain Lake CH4 Emissions – Developing Optimized Approaches to Representative Flux Measurements
Source: PLoS One. 2015 Apr 10;10(4):e0123319. doi: 10.1371/journal.pone.0123319 (PMC4393096; doi:10.1371/journal.pone.0123319)
Supplement: S1 Table — describe the CH4 emission (mmol m-2 d-1) with respectively day, position and transect within lake. See Fig 1 in methods section for an illustration of chambers placements. (PDF) [file pone.0123319.s001.pdf]

Supporting Information

Spatial versus day-to-day within-lake variability in tropical floodplain lake CH_4 emissions – developing optimized approaches to representative flux measurements

Roberta B. Peixoto[1*], Fausto Machado-Silva[1], Humberto Marotta[2], Alex Enrich-Prast[1,3], David Bastviken[3]

[1] Biogeochemistry laboratory, Department of Ecology, Universidade Federal do Rio de Janeiro, 68020, Rio de Janeiro, Brazil.

[2] Sedimentary and Environmental Processes Laboratory (LAPSA/UFF), Department of Geography, Institute of Geosciences, Universidade Federal Fluminense, Niteroi, Brazil.

[3] Department of Thematic Studies - Environmental Change, Linköping University, 58183, Linköping, Sweden.

*Corresponding author

This support information contains four pages and one table.

S1 Table: Data of methane emission from water to atmosphere.

Day	Sampling station	Transect	Total CH_4 emissions from lakes to the atmosphere (mmol m^{-2} d^{-1})
9/14/2008	WBM 1	a	2.72
9/14/2008	WBM 2	a	2.57
9/14/2008	WBM 3	a	3.52
9/14/2008	MOW 2	a	2.34
9/14/2008	MOW 3	a	2.44
9/14/2008	MOW 4	a	5.58
9/14/2008	WBM 1	b	3.75
9/14/2008	WBM 2	b	2.21
9/14/2008	MOW 1	b	1.23
9/14/2008	MOW 2	b	0.39
9/14/2008	MOW 3	b	0.87
9/14/2008	MOW 4	b	0.13
9/14/2008	WBM 1	c	1.52
9/14/2008	WBM 2	c	1.76
9/14/2008	WBM 3	c	2.17
9/14/2008	MOW 1	c	14.93
9/14/2008	MOW 2	c	2.58
9/14/2008	MOW 3	c	1.16

9/14/2008	MOW 4	c	1.20
9/14/2008	CEN 1		0.65
9/14/2008	CEN 2		2.67
9/14/2008	CEN 3		1.40
9/14/2008	CEN 4		0.43
9/15/2008	WBM 1	a	7.71
9/15/2008	WBM 2	a	3.93
9/15/2008	WBM 3	a	1.50
9/15/2008	MOW 1	a	4.31
9/15/2008	MOW 2	a	1.18
9/15/2008	MOW 3	a	7.65
9/15/2008	MOW 4	a	0.54
9/15/2008	WBM 1	b	8.55
9/15/2008	WBM 2	b	1.46
9/15/2008	WBM 3	b	1.97
9/15/2008	MOW 1	b	2.05
9/15/2008	MOW 2	b	1.85
9/15/2008	MOW 3	b	1.91
9/15/2008	MOW 4	b	1.36
9/15/2008	WBM 1	c	7.90
9/15/2008	WBM 2	c	9.76
9/15/2008	WBM 3	c	0.15
9/15/2008	MOW 1	c	1.81
9/15/2008	MOW 2	c	5.98
9/15/2008	MOW 4	c	1.18
9/15/2008	CEN 1		1.29
9/15/2008	CEN 2		1.04
9/15/2008	CEN 3		2.22
9/15/2008	CEN 4		1.18
9/16/2008	WBM 1	a	2.04
9/16/2008	WBM 2	a	6.01
9/16/2008	WBM 3	a	3.02
9/16/2008	MOW 1	a	8.06
9/16/2008	MOW 3	a	7.44
9/16/2008	MOW 4	a	1.18
9/16/2008	WBM 1	b	3.49
9/16/2008	WBM 2	b	3.74
9/16/2008	WBM 3	b	5.28
9/16/2008	MOW 1	b	1.50
9/16/2008	MOW 2	b	3.90
9/16/2008	MOW 3	b	1.87
9/16/2008	MOW 4	b	0.94
9/16/2008	WBM 1	c	3.12

9/16/2008	WBM 2	c	5.42
9/16/2008	WBM 3	c	0.87
9/16/2008	MOW 1	c	0.98
9/16/2008	MOW 2	c	3.00
9/16/2008	MOW 3	c	0.65
9/16/2008	MOW 4	c	2.03
9/16/2008	CEN 1		1.23
9/16/2008	CEN 2		1.68
9/16/2008	CEN 3		1.04
9/16/2008	CEN 4		0.46
9/17/2008	WBM 1	a	1.19
9/17/2008	WBM 3	a	11.04
9/17/2008	MOW 1	a	13.15
9/17/2008	MOW 2	a	10.27
9/17/2008	MOW 3	a	9.01
9/17/2008	MOW 4	a	1.79
9/17/2008	WBM 1	b	3.15
9/17/2008	WBM 3	b	3.56
9/17/2008	MOW 1	b	3.03
9/17/2008	MOW 2	b	4.69
9/17/2008	MOW 3	b	1.79
9/17/2008	MOW 4	b	0.47
9/17/2008	WBM 2	c	16.84
9/17/2008	WBM 3	c	10.83
9/17/2008	MOW 1	c	1.02
9/17/2008	MOW 2	c	2.57
9/17/2008	MOW 3	c	3.82
9/17/2008	MOW 4	c	0.70
9/17/2008	CEN 1		1.54
9/17/2008	CEN 2		2.25
9/17/2008	CEN 3		2.63
9/18/2008	WBM 1	a	3.31
9/18/2008	WBM 2	a	8.70
9/18/2008	WBM 3	a	1.62
9/18/2008	MOW 1	a	7.95
9/18/2008	MOW 2	a	7.63
9/18/2008	MOW 3	a	5.67
9/18/2008	MOW 4	a	1.90
9/18/2008	WBM 1	b	12.01
9/18/2008	WBM 2	b	8.95
9/18/2008	WBM 3	b	8.55
9/18/2008	MOW 1	b	4.63
9/18/2008	MOW 2	b	4.71

9/18/2008	MOW 3	b	3.87
9/18/2008	WBM 1	c	3.89
9/18/2008	WBM 2	c	11.47
9/18/2008	WBM 3	c	9.36
9/18/2008	MOW 1	c	0.76
9/18/2008	MOW 2	c	7.60
9/18/2008	MOW 3	c	1.64
9/18/2008	MOW 4	c	2.19
9/18/2008	CEN 1		2.39
9/18/2008	CEN 2		1.74
9/18/2008	CEN 3		3.04
9/18/2008	CEN 4		1.81

S1 table describe the CH_4 emission (mmol m^{-2} d^{-1}) with respectively day, position and transect within lake. See Fig. 1 in methods section for an illustration of chambers placements.
